# Carcinoma of gall bladder presenting as dermatomyositis

**DOI:** 10.4103/0972-2327.78050

**Published:** 2011

**Authors:** Deepti Akkihebbal Narasimhaiah, Jennifer Anne Premkumar, Viju Moses, Geeta Chacko

**Affiliations:** Department of General Pathology, Christian Medical College, Vellore- 632004, Tamil Nadu, India; 1 Department of Medicine, Christian Medical College, Vellore- 632004, Tamil Nadu, India; 2 Department of Neurosciences, Christian Medical College, Vellore- 632004, Tamil Nadu, India

**Keywords:** Dermatomyositis, carcinoma, gall bladder, paraneoplastic

## Abstract

Cancer-related muscle diseases are usually paraneoplastic disorders. Dermatomyositis (DM) is a type of inflammatory myopathy that is strongly associated with a broad range of malignant disorders. The malignancy can occur before, concomitantly or after the onset of myositis. The malignancies most commonly associated with DM are carcinomas of ovary, lung, stomach, colorectal and pancreas, as well as non-Hodgkin’s lymphoma. An association of DM with carcinoma of the gall bladder (GB) is extremely rare with only two previously reported cases in the literature. We report a case of carcinoma of GB with DM as the paraneoplastic manifestation.

## Introduction

Dermatomyositis (DM) is a type of inflammatory myopathy that is associated with a broad range of malignant disorders.[1] The cancers most strongly associated with DM are ovarian, lung, colorectal carcinomas and non-Hodgkin’s lymphoma.[2] An association of DM with carcinoma of the gall bladder (GB) is extremely rare with only two previously reported cases in the literature.[3,4] We report a case where DM was the primary manifestation of carcinoma of the GB.

## Case Report

A 65 year old woman presented with complaints of an erythematous rash involving the face and upper limbs and severe progressive proximal weakness since 2 months, dysphagia and nasal regurgitation since 1 month. Clinical examination revealed extensive DM rash with heliotrope rash, V sign and shawl sign; and the patient had severe proximal myopathy with bulbar weakness. A clinical diagnosis of DM was considered and the following investigations were performed for the confirmation of the same.

The hemogram and erythrocyte sedimentation rate were normal. The biochemical parameters–blood glucose, serum creatinine, blood urea, calcium and phosphate were within normal limits.

The liver function tests showed elevated aminotransferases. The alkaline phosphatase level was normal.

There was marked elevation of serum creatine kinase (CK) and lactate dehydrogenase, which were 1287 and 1432 U/L, respectively. The serum antinuclear antibody (ANA) showed weak positivity. Tests for lupus anticoagulant, rheumatoid factor and autoantibodies CENP, anti-RNP, Jo-1 and SCL-70 were negative. Serum complement, double-stranded DNA and anticardiolipin were within normal limits. The serum levels of cancer antigen 125 (CA-125) and carcinoembryonic antigen (CEA) were 49.9 U/mL (normal 0–21) and 31 ng/mL (normal < 5), respectively. Needle electromyography did not show any spontaneous activity.

The abdominal ultrasound revealed a soft tissue echogenicity lesion in the region of anterior wall of the fundus of GB with extension into the soft tissue, associated cholelithiasis and a subcentimeter hypoechoic lymph node at the porta hepatis. The rest of the abdominal viscera were normal. Based on these sonographic features, the possibility of GB malignancy was considered.

**Figure 1:**
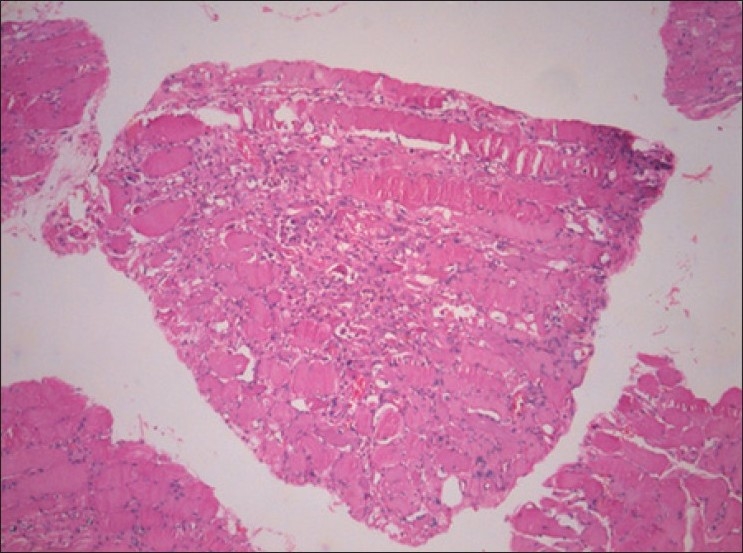
Vastus lateralis showing myopathic features (H and E, ×100)

**Figure 2:**
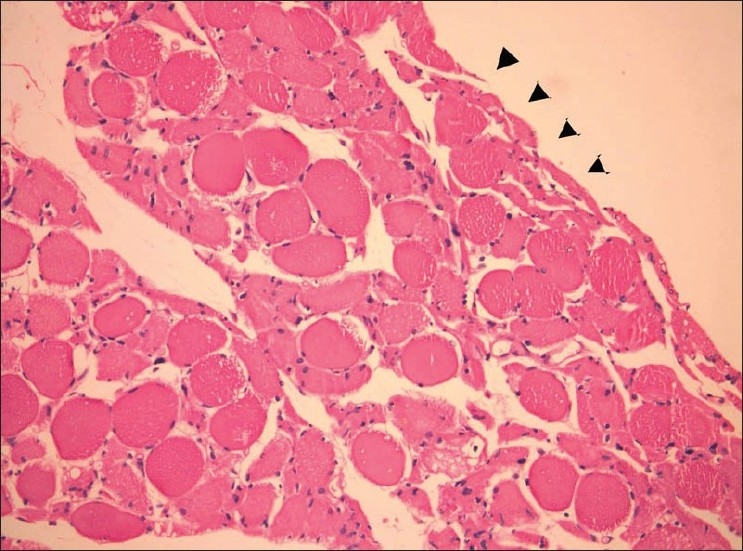
Atrophic fibers at the periphery of the fascicle (perifascicular atrophy–indicated by arrowheads) (H and E, ×200)

A biopsy from the right vastus lateralis showed myopathic features with fiber size variation, myophagocytosis and regenerative activity [Figure 1]. There was myofiber necrosis, perifascicular atrophy [Figure 2] and patchy endomysial infiltrate of lymphocytes. Occasional endomysial capillaries showed deposits of membrane attack complex of complement. The morphologic features were consistent with DM.

After the confirmation of a diagnosis of DM, she was started on immunosuppression with which there was improvement in muscle power. During her stay in the hospital, while awaiting further treatment for the GB carcinoma, she developed deep vein thrombosis of the left lower limb followed by an acute pulmonary thromboembolism and aspiration pneumonia. She had a cardiorespiratory arrest and expired despite resuscitative measures.

A partial autopsy of the thoracoabdominal viscera was performed after obtaining informed consent from the next of kin. At autopsy, there was a polypoidal growth in the fundus of the GB measuring 4 × 3.2 × 1.8 cm with a gray-white, firm cut surface. The rest of the mucosa did not reveal any abnormality on gross examination. There were two enlarged lymph nodes at the porta hepatis and the neck of the GB with tan, firm cut surfaces. The right lung showed consolidation of the lower lobe. There was no evidence of thrombi within the pulmonary vessels. The rest of the viscera did not reveal any abnormality on gross examination.

**Figure 3:**
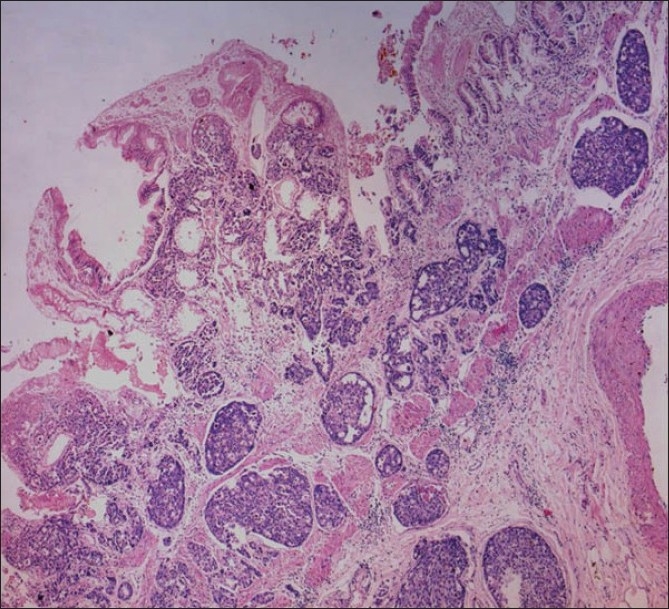
Tumor transmurally infiltrating the wall of gall bladder (H and E, ×50)

The microscopic examination of the tumor in GB showed a transmurally infiltrating moderately differentiated adenocarcinoma [Figure 3]. The tumor showed perineural invasion, as well as extension into the adventitial adipose tissue. The enlarged lymph nodes at the porta hepatis and the neck of GB showed metastatic tumor deposits. The right lung revealed lobar pneumonia with evidence of aspiration. The microscopic examination of the rest of the organs was normal.

Based on these findings, a diagnosis of adenocarcinoma of GB with lymph node metastasis was made at autopsy.

This case was unique in that the patient presented with DM as a paraneoplastic manifestation of the underlying GB carcinoma.

## Discussion

There are reports of an overall increased incidence of malignant disease in both men and women with idiopathic inflammatory myopathies, with the risk being highest in the first 3 years after the diagnosis of myositis.[5] The prevalence of malignancy in patients with inflammatory myopathy has been reported in the range of 4%–42%.[5] Both polymyositis (PM) and DM are associated with an increased risk of malignancy, with the risk being higher for DM.[2] The malignancy can occur before, concomitantly, or after the onset of myositis.[1] In the present case, the carcinoma of GB was present at the time of diagnosis of DM, but it was the occurrence of DM that prompted a search for the associated malignancy.

The mean age of patients with inflammatory myopathy at the time of diagnosis of malignancy has been reported to be 50.1 ± 12.6 years[6] with females being preferentially affected in DM associated with neoplasia.[1,4] In our case, the patient was a 65-year-old female. For typical DM or PM, no specific morphologic features have been identified that are predictive of an associated neoplasm.[7] The heliotrope rash may not be present, but periorbital edema is usually present.[4] In the present case, there was extensive DM rash. In the study by Lee *et al*., the mean level of serum CK was significantly lower in the malignancy group (1412.5 ± 568.6) than in the nonmalignancy group (3203.8 ± 585.5).[6] The CK in our case was 1287 U/L. However, the CK may remain normal in chronic cases.[4] In addition, the muscle biopsy that is considered as the gold standard may not yield any results because of patchy involvement.[4] In the present case, the features on muscle biopsy were diagnostic of DM. The etiology of DM and PM is unclear, but autoimmunity is considered to play a role in view of the presence of autoantibodies, which are known as myositis-specific or myositis-associated autoantibodies (MSAs/MAAAs). These autoantibodies are directed against the cytoplasmic and nuclear components and include antisynthetases: anti-Jo-1, anti-PL-7, anti-PL-12, anti-EJ, anti-OJ, anti-KS, anti-Mi-2,anti-SRP, anti-PM-Scl, anti-Ku, anti-U1-RNP, and anti-U3-RNP.[8,9] Apart from these, novel MSAs have been characterized, such as the anti-155/140 antibody, antibody directed against the small ubiquitin-like modifier-activating enzyme (SAE) and anti-MJ.[9–11] The anti-155/140 antibody is an autoantibody reactive against 155 and 140-kDa nuclear proteins, which is proposed to target the transcriptional intermediary factor 1-γ (TIF-Iγ)[8] and is frequently present in patients with DM associated with malignancy.[8,9] Hence, the addition of anti-155/140-kDa nuclear proteins would considerably aid the prediction of cancer-associated myositis. However, routine testing for anti-155/140-kDa nuclear proteins is currently not possible in view of the nonavailability of a commercially viable test for anti-155/140-kDa antibody.[8] The fact that TIF-1γ belongs to the family of proteins that are involved in cellular differentiation could further aid in elucidating the link between cancer and autoimmunity in myositis.[11] According to Casciola-Rosen *et al*, the myositis autoantigens are highly expressed in the myositis muscle within the regenerating muscle cells and the antigenic fingerprint of tumors associated with myositis strongly resembles that of the regenerating muscle cells in myositis. The development of an anticancer immune response directed against the autoantigens on the tumor cells could possibly trigger a nonspecific muscle injury leading to increased numbers of regenerating cells that express high levels of myositis autoantigens, which they share with the tumor cells thereby making them the target of antigen-specific immune responses leading to further muscle damage.[12]

One of the aspects of the association of rheumatic diseases with cancer is the development of musculoskeletal symptoms and syndromes as paraneoplastic manifestation. Paraneoplastic syndromes occur in association with neoplasms that lie anatomically remote from them. There are multiple mechanisms involved in the pathogenesis of the paraneoplastic musculoskeletal syndrome, such as (a) the production of ectopic humoral factors or proteins, including hormones, cytokines, peptides, or others, that may affect the musculoskeletal system; (b) altered immune surveillance; and (c) the role of several autoantibodies, such as antibodies to double-stranded DNA and ANAs.[7,13,14] Even though DM can be associated with a broad range of malignant disorders, an association of DM with tumors of the biliary tract is extremely rare. However, there are occasional reports of DM in patients with hepatocellular carcinoma and GB carcinoma.[3,4,15] The protocol for cancer screening in patients with DM is evolving. Clinical examination, radiologic imaging and tumor markers are commonly used. Newer modalities, such as integrated positron emission tomography is being evaluated in this area.

This case report indicates that GB carcinoma should be added to the list of malignancies associated with DM and has to be excluded by relevant investigations in elderly women presenting with inflammatory myopathies.

## References

[1] Kim HI, Chung SH, Hwang JE, Kim SH, Ahn JS, Yang DH (2007). Dermatomyositis associated with cancer of unknown primary site. J Korean Med Sci.

[2] Hill CL, Zhang Y, Sigurgeirsson B, Pukkala E, Mellemkjaer L, Airio A (2001). Frequency of specific cancer types in dermatomyositis and polymyositis: a population-based study. Lancet.

[3] Yiannopoulos G, Ravazoula P, Meimaris N, Stavropoulos M, Andonopoulos AP (2002). Dermatomyositis in a patient with adenocarcinoma of the gall bladder. Ann Rheum Dis.

[4] Kundu AK, Karmakar PS, Bera AB, Pal SK (2005). Carcinoma of the gall bladder presenting as dermatomyositis. JAPI.

[5] Buchbinder R, Forbes A, Hall S, Dennett X, Giles G (2001). Incidence of malignant disease in biopsy proven inflammatory myopathy - A population based cohort study. Ann Intern Med.

[6] Lee SW, Jung SY, Park MC, Park YB, Lee SK (2006). Malignancies in Korean patients with inflammatory myopathy. Yonsei Med J.

[7] Mozaffar T, Pestronk A (2002). Cancer related muscle disease. Structural and Molecular basis of skeletal muscle diseases.

[8] Chinoy H, Fertig N, Oddis CV, Ollier WE, Cooper RG (2007). The diagnostic utility of myositis autoantibody testing for predicting the risk of cancer-associated myositis. Ann Rheum Dis.

[9] Kaji K, Fujimoto M, Hasegawa M, Kondo M, Saito Y, Komura K (2007). Identification of a novel autoantibody reactive with 155 and 140 kDa nuclear proteins in patients with dermatomyositis: an association with malignancy. Rheumatology.

[10] Betteridge Z, Gunawardena H, North J, Slinn J, McHugh N (2007). Identification of a novel autoantibody directed against small ubiquitin-like modifier activating enzyme in dermatomyositis. Arthritis Rheum.

[11] Gunawardena H, Betteridge ZE, McHugh NJ (2008). Newly identified autoantibodies: relationship to idiopathic inflammatory myopathy subsets and pathogenesis. Curr Opin Rheumatol.

[12] Casciola-Rosen L, Nagaraju K, Plotz P (2005). Enhanced autoantigen expression in regenerating muscle cells in idiopathic inflammatory myopathy. JEM.

[13] András C, Csiki Z, Ponyi A, Illés A, Dankó K (2006). Paraneoplastic rheumatic syndromes. Rheumatol Int.

[14] Szekanecz E, András C, Sándor Z, Antal-Szalmás P, Szántó J, Tamási L (2006). Malignancies and soluble tumor antigens in rheumatic diseases. Autoimmun Rev.

[15] Toshikuni N, Torigoe R, Mitsunaga M, Omoto A, Nakashima K (2006). Dermatomyositis associated with hepatocellular carcinoma in an elderly female patient with hepatitis C virus-related liver cirrhosis. World J Gastroenterol.

